# Increase of respiratory illnesses among children in Beijing, China, during the autumn and winter of 2023

**DOI:** 10.2807/1560-7917.ES.2024.29.2.2300704

**Published:** 2024-01-11

**Authors:** Cheng Gong, Fang Huang, Luodan Suo, Xuejiao Guan, Lu Kang, Hui Xie, Geng Hu, Peng Yang, Quanyi Wang

**Affiliations:** 1 Beijing Center for Disease Prevention and Control, Beijing, People’s Republic of China; 2 Beijing Research Center for Respiratory Infectious Diseases, Beijing, People’s Republic of China; 3 These authors contributed equally to this study

**Keywords:** *Mycoplasma pneumoniae*, respiratory illness, surveillance, seasonality, pediatric pneumonia

## Abstract

In 2023, through an ongoing respiratory pathogen surveillance system, we observed from mid-September onwards, an increase of respiratory illness among children aged ≤ 15 years presenting at hospital outpatient clinics in Beijing, China. Data indicated that illness was caused by multiple pathogens, predominantly *Mycoplasma pneumoniae*. Seasonality, periodicity and high prevalence of resistance to macrolide (30 of 30 strains sequenced with the A2063G mutation) were important characteristics of the *M. pneumoniae* epidemic, which resulted in a rise in consultations at specialised paediatric hospitals.

Since mid-October of 2023, a marked increase in incidence of respiratory diseases has been noted in Northern China compared to the same period over the past 3 years [1]. Already in mid-September, the increase was also observed in Beijing, which, with a permanent population of 21 million, is the biggest city in the region. Since 2014, Beijing has benefited from a stable year-round respiratory pathogen surveillance system [2]. This allowed us to investigate and characterise the epidemic in the city.

## The respiratory pathogen surveillance system

In 2023, the respiratory pathogen surveillance system in Beijing consisted of 35 sentinel hospitals distributed across 16 districts and was supported by 17 collaborating laboratories coordinated by the Beijing Center for Disease Prevention and Control [3,4].

The numbers of outpatient consultations for acute respiratory tract infections (ARTIs), which include influenza-like illness (ILI) and pneumonia cases were recorded by the sentinel hospitals and reported weekly to the surveillance system. ARTI was defined as at least one acute-infection manifestation (e.g. fever, hypothermia, leucocytosis, leucopoenia) and one respiratory manifestation (e.g. sore throat, cough, sputum production, chest ache). ILI referred to World Health Organization (WHO)’s definition [5]. Pneumonia patients were included if they had evidence of community-acquired pneumonia according to the Guideline of Diagnosis and Treatment for Community-Acquired Pneumonia among Adults in China (released in 2016) [6].

Additionally, trained personnel at each hospital prospectively recruited 5–15 patients presenting with ARTI every week, employing a convenience sampling method. Among the total patients recruited, pneumonia cases constituted approximately 50% and children under 15 years old accounted for over 20%, regardless of sex. Respiratory specimens were collected either upon outpatient visits or within 3 days of hospitalisation, with no requirements on proportions of inpatients or outpatients to sample each week. Specimens were promptly transferred to the collaborating laboratories every week, where they were tested by real-time PCR for common respiratory pathogens, including enterovirus, human rhinovirus, human adenovirus, human bocavirus, human coronavirus, human metapneumovirus, influenza virus, parainfluenza virus, respiratory syncytial virus, as well as *Mycoplasma pneumoniae* and *Chlamydia pneumoniae*. Testing for severe acute respiratory syndrome coronavirus 2 (SARS-CoV-2) was also done, as this virus was added to the surveillance after China lifted its restrictions related to COVID-19 in December 2022. Genetic material from samples testing positive for *M. pneumoniae* was further subjected to real-time PCR to determine subtypes (P1-1 and P1-2) and sequenced to check for the A2063G mutation. Laboratory results were reported to the respiratory pathogen surveillance system. The positivity rate for respiratory pathogens was determined by dividing the number of specimens testing positive for any respiratory pathogen by the total number of cases recruited from the 35 sentinel hospitals every month. Of note, aside from a few, further described, interruptions in some months, due to surveillance being temporarily suspended when strict public health measures were applied during the COVID-19 pandemic, ARTI cases have been recruited in the same way since 2019.

The findings of the respiratory pathogen surveillance system were regularly communicated to all sentinel hospitals on a weekly basis.

## An increase in respiratory illnesses in Beijing

**Figure 1:**
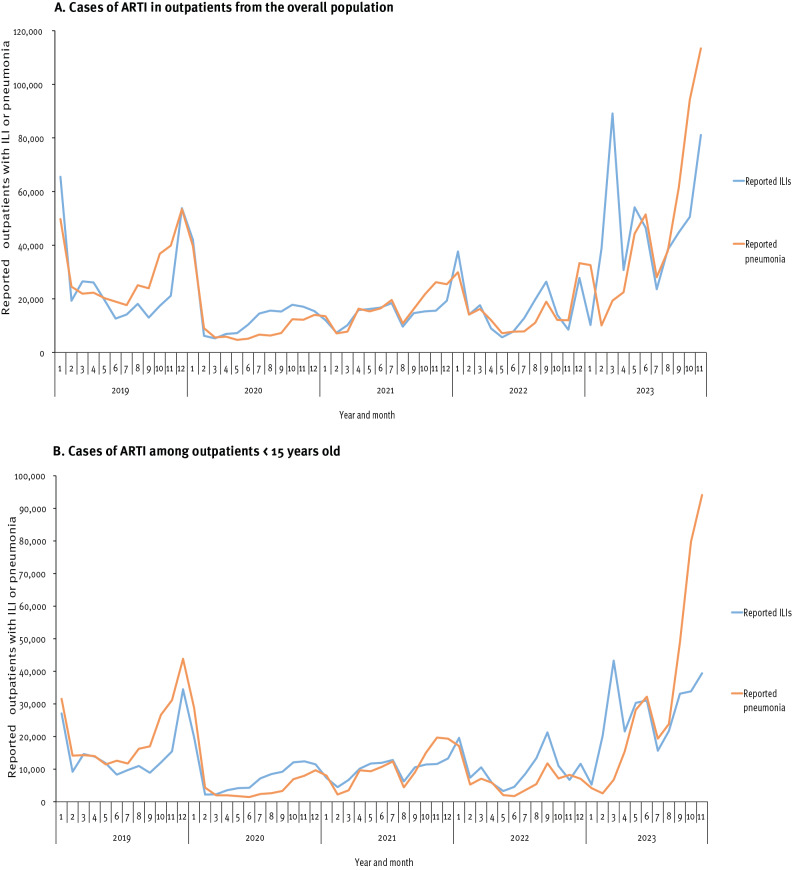
Monthly distribution of reported numbers of outpatients with acute respiratory tract infection, including influenza-like illness or pneumonia, in (A) the overall population and (B) children^a^, Beijing, China, 2019–2023 (n = 1,689,123 patients)

Our surveillance data indicate an increase in consultations for ILI and pneumonia cases among the general population starting from September 2023. This increase was particularly notable during October and November, with a rise for ILI of 81.2% (n = 81,091 patients in November) and for pneumonia of 84.5% (n = 113,394 patients in November) respectively, compared to September (n = 44,740 ILI patients and n = 61,471 pneumonia patients). These October–November levels in 2023 also surpassed those of the previous 4 years but not those of ILI in early 2023 (Figure 1A). Among children under 15 years old, there was a more considerable rise in pneumonia cases from September to November, compared to ILI cases (Figure 1B), with numbers of pneumonia cases ascending from 48,921 to 94,099 in these months, corresponding to a 92.3% increase, whereas numbers of ILI cases only rose by 18.9% (33,142 in September vs 39,417 in November).

ARTI: acute respiratory tract infection.

^a^ Children are defined as people under 15 years old.

## Co-circulation of multiple respiratory pathogens including *Mycoplasma pneumoniae*

**Figure 2:**
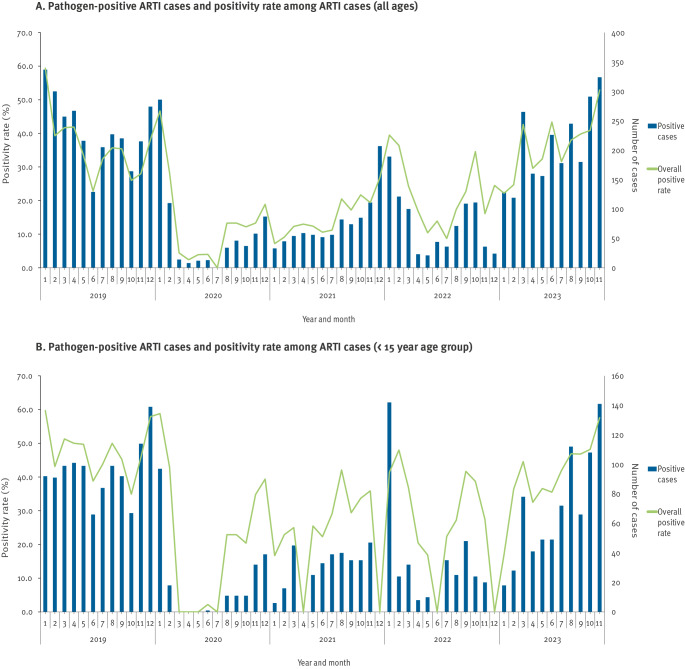
Monthly distribution of recruited cases of acute respiratory tract infection, who tested positive for a respiratory pathogen^a^ and overall respiratory pathogen detection rate among recruited cases (A) of all ages and (B) < 15 years old, Beijing, China, 2019–2023 (n = 11 respiratory pathogens)^a^

The positivity rate for respiratory pathogens detected among the recruited ARTI cases of all ages in 2023 was substantially higher than during the September to November periods of the 3 prior COVID-19-pandemic years (Figure 2A). For children, the positivity rate during these months in 2023 was also more elevated compared to each September to November period in the past 3 years (Figure 2B).

ARTI: acute respiratory infection.

^a^ Respiratory pathogens include any of the following: *Chlamydia pneumoniae,* enterovirus, human adenovirus, human bocavirus, human coronavirus including NL63, OC43, 229E and HKU1, human metapneumovirus, influenza virus, *Mycoplasma pneumoniae*, parainfluenza virus, human rhinovirus and respiratory syncytial virus.

**Figure 3:**
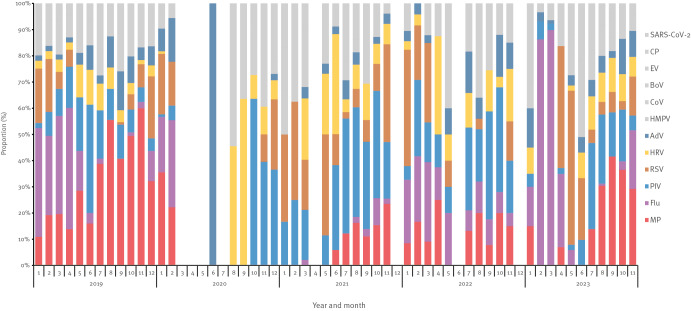
Monthly proportions of respiratory pathogens among children under 15 years old, Beijing, China, 2019–2023 (n = 12 respiratory pathogens)

AdV: human adenovirus; BoV: human bocavirus; CoV: human coronavirus including NL63, OC43, 229E and HKU1; CP: *Chlamydia pneumonniae*; EV: enterovirus; Flu: influenza virus; HMPV: human metapneumovirus; MP: *Mycoplasma pneumoniae*; PIV: parainfluenza virus; RV: human rhinovirus; RSV: respiratory syncytial virus; SARS-CoV-2: severe acute respiratory syndrome coronavirus 2.

Our surveillance data revealed that the rise in incidence of respiratory diseases among children between September and November 2023 in Beijing coincided with the increased positivity rate of common respiratory pathogens. *M. pneumoniae*, parainfluenza virus and human rhinovirus predominated in September, *M. pneumoniae*, parainfluenza virus and human adenovirus in October, and *M. pneumoniae*, influenza virus and respiratory syncytial virus in November (Figure 3). This finding strongly suggests that the rise in respiratory illnesses among the children in Beijing was directly linked to well-known respiratory pathogens that were circulating. It aligns with the recent risk assessment by the World Health Organization (WHO), which also underscored the cause for the escalation of respiratory illnesses among children in Northern China as known pathogens [7].

No surveillance data are shown in some months over past years (especially in first half of 2020) because surveillance was temporarily suspended when strict public health measures were applied during the COVID-19 pandemic.

**Figure 4:**
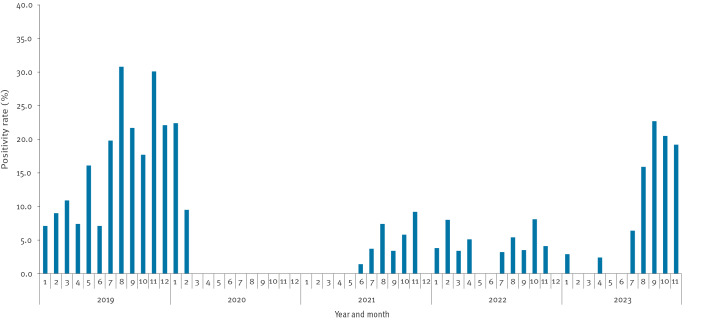
Distribution of proportion of *Mycoplasma pneumoniae* positive samples among children less than 15 years old detected by the surveillance system, Beijing, China, 2019–2023

It is important to highlight that *M. pneumoniae* played a crucial role in the rise of respiratory illnesses among children during the autumn/winter of 2023, exhibiting longer circulation and resulting in higher proportions of infections compared to other respiratory pathogens. Through our surveillance, we prior observed a substantial *M. pneumoniae* epidemic between late 2019 and early 2020, coinciding with simultaneous epidemics across multiple countries, particularly in Europe and Asia (Figure 4) [8]. However, *M. pneumoniae* circulation was extremely limited from 2020 to 2022 since strict public health measures against COVID-19 were adopted in Beijing on 24 January 2020, which were only lifted in December 2022.

Our surveillance found that among a total of 100 children’s specimens positive for* M. pneumoniae* in 2023, genotype P1-1 predominated, accounting for 74% of the strains, which was higher than 60.6% (238/393) in 2019 and the combined rate of 43.1% (43/88) in 2021 and 2022, but lower than the 83.7% (41/49) recorded in 2020. 

The ribosomal RNA (rRNA) in 30 of the *M. pneumoniae* samples from 2023, including 20 strains of genotype P1-1 and 10 of P1-2, was sequenced. This revealed that all 30 strains carried the A2063G mutation in the V domain of rRNA gene, indicating that they were macrolide resistant, an issue which has been an ongoing in recent years [4]. Cao et al*.* reported that the minimum inhibitory concentration (MIC) for 112 *M. pneumoniae* isolates with the A2063G mutation ranged from 2 to 32 μg/mL for azithromycin and from 128 to ≥ 256 μg/mL for erythromycin, considering MIC resistance thresholds of ≥ 2 μg/mL and ≥ 32 μg/mL for these two macrolides respectively [9]. Macrolide antibiotics, such as azithromycin, are the preferred treatment for *M. pneumoniae* pneumonia (MPP) in China. Tetracycline antibiotics, such as doxycycline and minocycline, are the primary alternative medications for treating MPP, particularly in cases of refractory MPP, macrolide-unresponsive MPP, and severe MPP. Considering the potential side effects of tooth discoloration and impaired enamel development, doxycycline and minocycline are only recommended for children aged 8 years and above [10].

## Discussion

Our surveillance indicates that the *M. pneumoniae* epidemic in 2023 still followed a characteristic seasonal pattern, by circulating during the autumn and winter seasons. The interval between the last epidemic in 2019 and the current one was approximately 4 years, which in principle is consistent with the periodic nature of *M. pneumoniae* epidemics that have a recognised cycle interval of 1–3 years [11]. The periodicity of an infectious disease can be largely attributed to the accumulation of a susceptible population and the subsequent decline in population immunity, which occurs due to relatively low infection levels during the inter-epidemic period [8]. The implementation of restriction measures during the COVID-19 pandemic may have theoretically amplified this effect. Based on a global *M. pneumoniae* surveillance study that covered 24 countries across four continents, Sauteur et al. also reported the delayed re-emergence of *M. pneumoniae* epidemics due to the introduction of non-pharmaceutical intervention measures against COVID-19 [8]. Additionally, similar to our study, during the current winter season, a rise in *M. pneumoniae* infections across all age groups was observed in several European countries, with a higher prevalence observed among children and adolescents [12]. This increase followed a 3-year period of minimal transmission in conjunction with widespread implementation of non-pharmaceutical measures during the COVID-19 pandemic, which possibly led to a decline in population immunity. It is noteworthy that no atypical *M. pneumoniae *strains or first-line macrolide antibiotic resistance were reported, which was consistent to the finding of European Centre for Disease Prevention and Control [12].

In addition to the seasonal and periodic factors likely contributing to the increase in respiratory illnesses among children in China, the high prevalence of resistance of *M*. *pneumoniae* to macrolide antibiotics, especially for azithromycin, may have also been an important factor. The inadequate effectiveness of treatment and the absence of alternative effective drugs for children under 8 years old might have led to a persisting infectious state, resulting in repeated visits by the same patients to clinical settings. This situation may have potentially increased the risk of the pathogen spreading.

The accumulation of unrecovering patients, along with the occurrence of new cases, may have contributed to the rise of consultations at specialised paediatric hospitals, placing a substantial strain on healthcare resources. Other factors, such as the lack of rapid and convenient assays for common respiratory pathogens in primary healthcare institutions, along with the significant imbalance in patient visits between primary healthcare institutions and specialised paediatric hospitals (data not shown), might have impacted on the supply of paediatric medical resources.

Our surveillance data provide profound insights into the causes of the increased prevalence of respiratory infections among children in the current winter season in Northern China. They highlight that, not only novel respiratory pathogens, but also well-known common pathogens can pose significant stain on healthcare in densely populated cities, especially megacities.

This study has nevertheless some limitations. First, outpatient case data were utilised to depict the rise of respiratory illnesses in Beijing during the autumn and winter of 2023. However, the actual number within the community was not estimated. Second, ARTI cases were recruited in a convenience sampling with 50% being pneumonia, which could have introduced a selection bias. Third, *M. pneumoniae* isolates were exclusively screened for gene mutations associated with macrolide resistance, while the phenotypical assessment of drug resistance was not performed.

Comprehensive measures and proactive strategies are warranted for future pandemics and extreme epidemic situations caused by both novel and known respiratory pathogens, particularly to alleviate the strain on healthcare resources. These measures include strengthening both etiological surveillance and syndromic surveillance, implementing non-pharmaceutical interventions among high-risk population groups, increasing coverage for existing vaccines against common respiratory pathogens, and introducing new respiratory vaccines, developing innovative antibiotics, improving the laboratory capacities of primary healthcare institutions, optimising tiered diagnosis and treatment policies, and expanding internet-based clinical services. Such efforts are crucial to ensure effective preparedness and response in the face of potential healthcare challenges.
